# Ethical considerations for teaching with artificial intelligence: a scoping review in medical education settings

**DOI:** 10.1186/s41239-025-00563-9

**Published:** 2025-11-17

**Authors:** Amani Itani, Susie L. Gronseth, Salma Musaad, Thuy Nguyen, Yiming Mirabile, Bettina M. Beech

**Affiliations:** 1 Department of Curriculum and Instruction, University of Houston, 3623 Cullen Blvd., Room 2030 McElhinney Hall, Houston, TX 77204-5023 USA; 2 Children’s Nutrition Research Center, Baylor College of Medicine, Houston, USA; 3 UH Population Health, University of Houston, Houston, USA

**Keywords:** Medical education, Artificial intelligence, AI in education, AIED, AI ethics, Curriculum development, ChatGPT, Scoping review

## Abstract

In the evolving realm of medical education, where artificial intelligence (AI) technologies play an increasingly pivotal role, embedding AI ethics within educational frameworks has become crucial. This scoping review explores the breadth of literature concerning ethical considerations for teaching with AI in medical education settings. Following the Preferred Reporting Items for Systematic Reviews and Meta-analyses extension for Scoping Reviews (PRISMA-ScR) guidelines, the team analyzed 82 peer-reviewed English-language articles published between 2018 and October 2023, sourced from PubMed, Embase, and Web of Science. The review presents insights into the geographic distribution of studies, publication trends, article types, common keywords, areas of focus, and ethical considerations. Ethical considerations are framed around the core principles of autonomy, beneficence, non-maleficence, justice, and transparency. Emerging themes and notable gaps are identified, offering suggested directions for future research. Review articles were found to be the most common study type, and “artificial intelligence” and “education” were the most frequently recurring keywords in the articles. The articles primarily focused on (a) incorporating AI in medical education, (b) AI ethical principles in medical education, and (c) ChatGPT in healthcare education. Ethical concerns raised in the articles centered on privacy, bias, informed consent, and algorithmic transparency, with many offering implications for curriculum development. These findings underscore the significance of integrating AI tools as instructional aids to enhance medical education, improve student learning experiences, and address the ethical challenges associated with their use in instructional settings. Future research opportunities include refining standardized competencies for AI in medical education, developing evidence-based curricula, and conducting longitudinal studies to assess AI’s long-term impact on learning outcomes.

## Introduction

The introduction of artificial intelligence (AI), particularly generative language models (GLMs), in education marks the beginning of a potentially transformative new era (Walter, 2024). Referred to as *AIEd*, artificial intelligence in education has been present and evolving in its capabilities to support education since around the 1980s (Russell & Norvig, 2010). With transformative developments in AI technologies happening rapidly, its revolutionary impacts on education are being recognized and prompting calls for enhancements to higher education teaching methods (Xia & Li, 2022). AI readiness implies understanding what AI is and what can be achieved with its use. Integrating AI in education is supported by educators’ AI-specific technological and pedagogical knowledge, which includes understanding the associated ethical issues (Celik, 2023).

In medical education settings, AI-enabled tools offer tremendous potential to enhance learning by generating novel content, developing simulations, creating digital patients, and providing real-time, personalized feedback and assessments. These capabilities enable students to engage in dynamic and realistic learning experiences that improve understanding and retention of medical concepts (Alam et al., 2023; Karabacak et al., 2023). ChatGPT can simulate patient interactions, assist with research and academic writing, generate case studies, and provide interactive tutorials that enhance student engagement and comprehension (Xu et al., 2024). Similarly, Dall-E, a graphical AI tool, can generate medical visuals such as anatomical diagrams, simulated X-rays, and ECG patterns that can be used in radiology and cardiology instruction (Amri & Hisan, 2023). Using AI in these ways helps students visualize complex medical concepts, assists them with formulating and discussing diagnoses, and reinforces their understanding interactively (Albahri et al., 2023). For instance, machine learning and deep learning facilitate interactive training in radiology by helping students analyze and interpret medical images in simulated environments (Xia & Li, 2022; Zhang et al., 2023). AI-based virtual labs allow students to explore anatomical structures in greater depth, while adaptive learning platforms personalize instruction by tailoring content to individual progress (Zhang et al., 2023).

Despite recognizing such affordances, educators are also grappling with ensuring safety and minimizing risks associated with AI use. Leading medical education organizations have increasingly stressed the importance of integrating AI into medical training responsibly and ethically. The American Medical Association (AMA, 2023) advocates for AI frameworks that prioritize transparency, equity, and accountability, ensuring that AI tools are used in ways that maintain trust. Likewise, the Association of American Medical Colleges (AAMC) has outlined key principles in ethical implementation, inclusive access, cross-disciplinary collaboration, and data security in medical education (AAMC, 2025). These efforts underscore the need for structured policies and ongoing oversight that maximizes the benefits of AI-powered learning while safeguarding fairness, privacy, and reliability.

Theoretical connections recommended by Jeffrey (2024) provide a lens for exploring the integration of AI into medical education through various ethical theories outlined in the literature. From a deontological perspective, students are expected to produce their authentic work, and using AI to create substantial portions of assignments would be considered unethical. Similarly, educators are responsible for ensuring knowledge is transferred ethically, and allowing students to rely on AI would undermine this duty. The notion of consequentialism is applied to evaluating the outcomes of AI use, suggesting that while it may save time, the potential harm to students’ learning outweighs any benefits. Further, care ethics emphasize the relational aspects of decision-making, indicating that actions involving AI must demonstrate care for all stakeholders. AI uses that negatively impact student learning or fail to respect the educator's role would conflict with this ethical framework.

Education can foster responsible innovation, centering the ethical guidelines for AI use in curriculum planning and the thoughtful incorporation of AI-driven tools (Xia & Li, 2022). At its foundation, AI ethics provides “a set of values, principles, and techniques that employ widely accepted standards of right and wrong to guide moral conduct in the development and use of AI technologies” (Leslie, 2019, p. 3). Ethical aspects can have significant implications for the development and application of AI in medical education settings (Dumić-Čule et al., 2020; Weidener & Fischer, 2023). Ethical considerations for integrating AI into medical education emphasize principles of autonomy, justice, non-maleficence, and beneficence (Busch et al., 2023) and raise concerns about bias, data privacy, security, fairness, informed consent, transparency, and accountability for how algorithms are developed and applied, as well as the accuracy of AI-generated content (Alam et al., 2023; Grunhut et al., 2022; Paranjape et al., 2019; Reddy, 2023). Ethically mindful use of AI involves pursuing models based on precise and dependable data and grounding decision-making in transparency and comprehensibility (Albahri et al., 2023).

Thus, research on ethical considerations of AI use in medical education has begun to emerge, with the field viewing the integration of AI as presenting “a critical juncture between transformative advancements and significant risks” (Knopp et al., 2023, p. 8). This scoping review addresses this need by exploring the ethical considerations for AI instructional applications in medical education settings. In the search process for this review, we framed the scope of medical education settings, defined by Swanwick (2019, p. 3), to include publications relating to “undergraduate, postgraduate, and the continuing professional development of established clinicians.” The purpose of the review is to examine literature published on AI ethics in medical education settings to provide an overview of characteristics, including geographic locations, journals, article types, publication years, keywords, focus areas, and ethical considerations. The review sought to identify gaps and challenges related to AI ethics in medical education that could inform a future research agenda and synthesize implications for curriculum development and instructional practices.

Two research questions guided this study:

1. How have publications on ethical considerations for teaching with AI in medical education settings developed over time?
2. What are the key ethical considerations highlighted in the literature on teaching with AI in medical education settings?

## Methods

A scoping review approach was chosen for this study as it is appropriate for identifying types of evidence currently available in the field, key characteristics of the research, and knowledge gaps in the body of literature (Munn et al., 2018). We used the Preferred Reporting Items for Systematic reviews and Meta-Analyses Extension for Scoping Reviews (PRISMA-ScR) checklist to guide our literature search (Tricco et al., 2018). The interdisciplinary research team spanned two academic institutions, with a doctoral student and faculty member from an academic research university and two statisticians and a faculty member from a medical school in the south-central United States. Following a consultation with a medical education librarian, we identified inclusion and exclusion criteria (as listed in Table 1), search terms, and keywords. Three databases (PubMed, Embase, and Web of Science) were selected for the search. An initial search scope spanning 2013 to 2023 was explored. Finding that results were concentrated mainly between 2018 and 2023, we narrowed our timespan to this period. The search was also limited to articles published in English.

**Table 1:** Inclusion and exclusion criteria

	Inclusion	Exclusion
1	Indexed in EMBASE, Web of Science, and PubMed and peer-reviewed	Not peer-reviewed articles, papers, book chapters, reports, conference proceedings
2	Ethical considerations for artificial intelligence use in education	No ethical considerations
		No artificial intelligence use
3	Medical education setting	No medical education setting
4	Published 2018 until October 2023	Published before 2018
5	English language	Not in English

### Search strategy

The search was completed over three months, from August to October 2023. Three research team members conducted the initial search step through the selected databases using the Medical Subject Headings (MeSH) terms and keywords (see Table 2). Potential articles were recorded in Microsoft Excel spreadsheets shared within the team.

**Table 2:** Search strings

Concept	Search string
Medical education	((Artificial intelligence) OR (machine learning)) AND (((medicine) OR (medical)) AND (education))) AND ((ethics) OR (ethical)) AND ((medical school education) OR (healthcare education))
ChatGPT	((Artificial intelligence) OR (machine learning)) AND (((medicine) OR (medical)) AND (education)) AND ((ethics) OR (ethical)) AND (ChatGPT)
AI health ethics	((Artificial intelligence) OR (machine learning)) AND (((medicine) OR (medical)) AND (education)) AND ((ethics) OR (ethical)) AND ((AIEd ethics) OR (health AI ethics))
Ethical challenges	((Artificial intelligence) OR (machine learning)) AND (((medicine) OR (medical)) AND (education)) AND ((ethics) OR (ethical)) AND ((artificial intelligence in education) OR (ethical challenges))

### Screening process

**Fig. 1:**
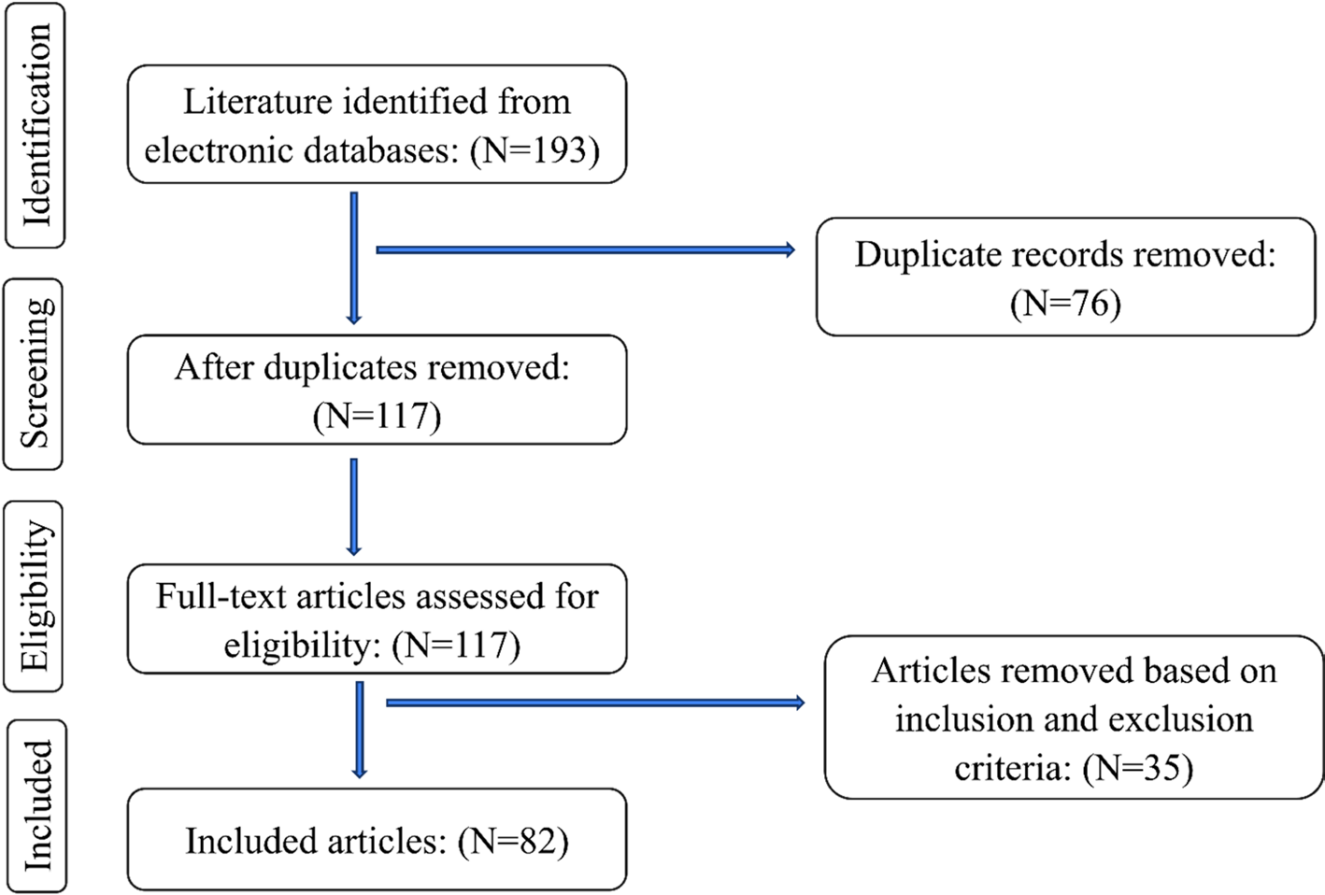
Literature Screening Process. Data collected by A. Itani, S. Gronseth, S. Musaad, T. Nguyen, and Y. Mirabile in 2023 from PubMed, Embase, and Web of Science

From an initial 193 database search results, three research team members screened the titles and abstracts of the identified articles. For each entry, the article number, search term, article title, article link, and database were recorded by the research team. Seventy-six duplicate records were identified and removed. The remaining 117 articles were independently double-screened by two members, and discrepancies were adjudicated through consensus in team meetings. Thirty-five articles deemed by the team as not meeting inclusion and exclusion criteria were removed, leaving 82 records for data extraction and synthesis (see Fig. 1).

### Data extraction and analysis

The team reviewed the full text of each article, extracting the following elements: author names, journal, publication year, article type, country of origin for first author affiliation, research aims and questions, keywords, results/findings, discussion/conclusion, and identified ethical concerns. The extracted data was first analyzed quantitatively using SAS programming language, with all variables maintained in character format.

After discussing the quantitative analysis within the team, a qualitative analysis phase of the results/findings and discussion/conclusion portions of the articles commenced. The coding process began with an open-ended exploration of the texts, identifying recurring patterns and ethical issues related to AI in medical education. A codebook (see Table 3) was generated based on observable patterns with nine main codes and associated sub-codes. The codebook was developed through an inductive approach, allowing themes and sub-themes to emerge directly from the analysis of the reviewed articles (Braun & Clarke, 2006). The codebook was used for subsequent coding rounds to ensure uniformity and reliability in the coding process. Two team members separately coded the results/findings and discussions/conclusions sections of the 82 included articles using the codebook. Two other team members then reviewed the coding and provided feedback via comments in a shared Microsoft Excel spreadsheet. The team reviewed and discussed the qualitative coding during team meetings, resolving any disagreements in the coding until a 100% consensus was reached.

**Table 3:** Codebook

Code	Sub-code
Incorporating AI in Medical Education	• Incorporating AI in psychology curriculum for mental healthcare • Incorporating AI in oral medicine diagnosis • Incorporating AI in anatomy education • Incorporating AI in medical physics education • Incorporating AI in radiology education • Incorporating AI in surgical education • Incorporating AI Generative Adversarial Networks (GANs) in medical education • AI and compassion in healthcare • Incorporating AI in pharmacy education
AI Ethical Principles in Medical Education	• Privacy concerns of students • AI ethics gap in the literature
ChatGPT in Healthcare Education	• ChatGPT for diabetes education • Incorporating AI in bioethics education
Evaluation of Medical AI Readiness	• AI readiness assessment for medical students
Challenges (and Opportunities) in AI Medical Education	• Challenges in including AI ophthalmology education
AI Competency	• Including digital competencies (DiCo) in education • AI competency guidance
AI Training in Medical Education	• Training radiology educators about AI
AI Student Knowledge/Perspectives	• AI student knowledge/perspectives • AI educator knowledge/perspectives
AI Case-Based Learning	• Learning from AI cases

## Results

### First-author locations

**Fig. 2:**
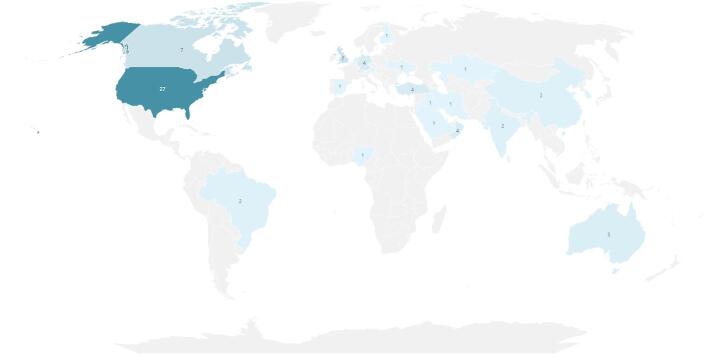
Distribution of Records by First-Author Affiliation Country of Origin. Data collected by A. Itani, S. Gronseth, S. Musaad, T. Nguyen, and Y. Mirabile in 2023 from PubMed, Embase, and Web of Science

Regarding the geographic distribution of identified publications based on the country of origin of the first author’s affiliation, a third of the articles (27) had US-based first-author affiliations (see Fig. 2). First-author affiliations in Canada and the UK accounted for approximately 9% each, having seven publications affiliated with each country. Germany, Oman, and Turkey each had four publications with first-author affiliations. Other country representations with less than 4% (i.e., three or fewer) of the corpus of articles included Australia, Austria, Belgium, Brazil, China, Finland, India, Iran, Iraq, Jordan, Kazakhstan, Netherlands, Nigeria, Qatar, Republic of Korea, Saudi Arabia, Singapore, Spain, Switzerland, and Ukraine.

### Journals

The articles were published across 50 journals in medical education, artificial intelligence, health sciences education, educational technologies, and ethics. Only 15 journals were associated with more than one article (see Table 4). The journals with the largest number of articles are *JMIR Medical Education* (10), *Academic Medicine* (5), *BMC Medical Education* (4), *Cureus* (4), and *Frontiers in Artificial Intelligence* (3). The remaining 36 articles were published in various other journals (as detailed in the complete list of articles provided in Appendix A). This diverse journal distribution highlights the interdisciplinary nature of AI ethics and suggests that ethical considerations for AI use in medical education are being examined across multiple domains.

**Table 4:** Journal frequencies with two or more articles

Journal	Count	Percentage (%)
JMIR Medical Education	10	12
Academic Medicine	5	6
BMC Medical Education	4	5
Cureus	4	5
Frontiers in Artificial Intelligence	3	4
Advances in Health Sciences Education	2	2
AMA Journal of Ethics	2	2
Anatomical Sciences Education	2	2
Artificial Intelligence in Medicine	2	2
International Journal of Artificial Intelligence in Education	2	2
International Journal of Environmental Research and Public Health	2	2
Medical Science Educator	2	2
Medical Teacher	2	2
PLoS Digital Health	2	2
Radiology: Artificial Intelligence	2	2

### Article type

Four article types were identified—editorial, empirical, review, and theoretical. The team also noted the sections in which the articles appeared. Review articles, constituting 37%, were the most prominent, reflecting a broad emphasis on synthesizing existing knowledge. In addition to appearing in designated “Review” sections, these narrative, critical, scoping, and systematic review articles were also found in “Educational Perspective,” “Descriptive,” “Conceptual Analysis,” and “Original Research” sections. Approximately 29% of the articles were empirical, appearing in “Original,” “Research,” “Education,” and “Scholarly Perspectives” sections. Editorial articles constituted about 28% and were in “Editorial,” “Letter to the Editor,” “Viewpoint,” “Reflections,” “Correspondence,” “Commentary,” and “Opinion” sections. The remainder were theoretical articles (6%), found in the “Target Article,” “Research Paper,” and “Perspective” sections.

### Publications by year

**Fig. 3:**
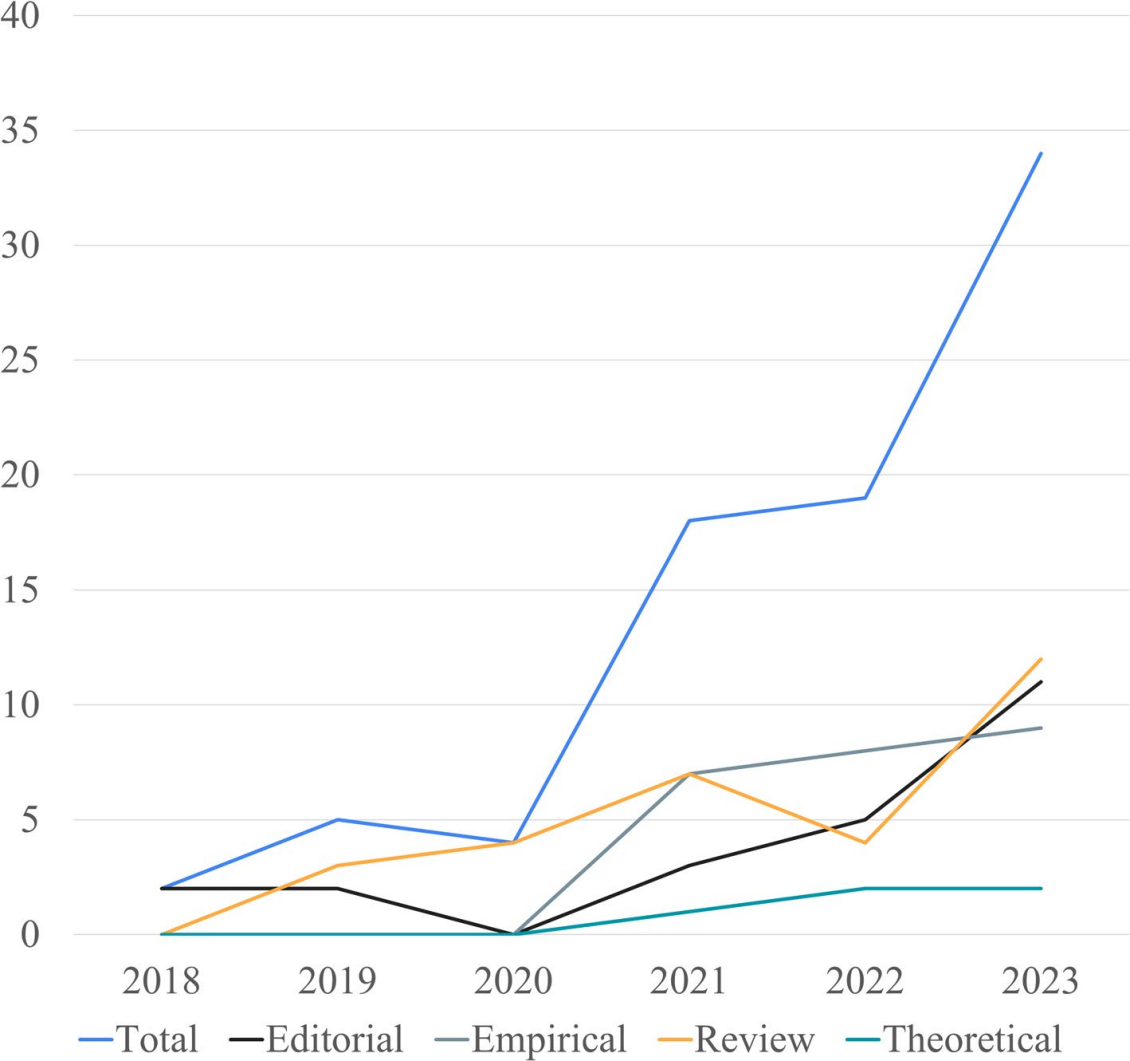
Articles Published by Year. Data collected by A. Itani, S. Gronseth, S. Musaad, T. Nguyen, and Y. Mirabile in 2023 from PubMed, Embase, and Web of Science

Figure 3 depicts the annual distribution of publications from January 2018 to October 2023. There were only two editorial articles in the first year of the review (2018). Approximately 87% of the articles were published between 2021 and 2023, with a substantial increase from 2022 to 2023 that doubled the publication rate. The significant rise in publications from 2021 to 2023 indicates increasing awareness of the ethical challenges associated with AI in medical education, likely spurred by the swift adoption of AI technologies like ChatGPT. Empirical, review, and editorial articles trended upward across the timespan. For example, review articles were present from 2019 onwards and increased fourfold. This shows that research during that time was primarily exploratory, focusing on synthesizing existing literature rather than conducting empirical studies. Furthermore, empirical articles emerged in the second half of the period. The theoretical articles' publication pattern was flat overall, with zero to two publications each year.

### Keywords

Out of the 65 articles that had keywords listed, the most frequently occurring keywords were “artificial intelligence,” “education,” “medical education,” “machine learning,” and “ChatGPT.” “Artificial intelligence” emerged as the most frequent keyword, constituting 74% of the 65 articles with a count of 48 keywords. “Education” was the next most frequent, appearing 45 times and contributing 69% to the overall article count. “Medical education” represented 48% of the articles, with 31 occurrences. “Machine learning” comprised 37% of the articles with 24 mentions. “ChatGPT” had 18% of instances, with 12 occurrences.

### Focus areas

From the qualitative analysis of the publications' research questions, aims, and discussion/conclusion sections, incorporating AI in medical education was identified as the most prevalent focus area (represented in 37% of the articles). The need for integrating AI ethical principles was the next prevalent focus area (26%), followed by ChatGPT in healthcare education (12%), evaluation of medical AI readiness (9%), and challenges and opportunities in AI medical education (7%). The remainder of the publications (9%) focused on AI competency, AI training in medical education, AI student knowledge/perspectives, and AI case-based learning.

### Focus areas and article types

Further exploring the focal areas by article type, articles on incorporating AI in medical education were most frequently published as review articles, followed by editorials (see Table 5). Articles focusing on AI ethical principles were in all four article types, with about a third being review articles, followed by editorials and then empirical studies. ChatGPT in healthcare education was almost evenly distributed across article types. Theoretical articles only focused on AI ethical principles in medical education, ChatGPT in healthcare education, and AI case-based learning.

**Table 5:** Frequency of focus areas according to article type

Focus areas	Article type				
	Total	Editorial *n*	Empirical *n*	Review *n*	Theoretical *n*
1. Incorporating AI in medical education	30	9	6	15	0
2. AI ethical principles in medical education	21	6	4	8	3
3. ChatGPT in healthcare education	10	2	3	4	1
4. Evaluation of medical AI readiness	7	0	5	2	0
5. Challenges (and opportunities) in AI medical education	6	5	0	1	0
6. AI competency	3	0	3	0	0
7. AI training in medical education	2	1	1	0	0
8. AI student knowledge/perspectives	2	0	2	0	0
9. AI case-based learning	1	0	0	0	1
Total	82	23	24	30	5

### Ethical considerations

**Fig. 4:**
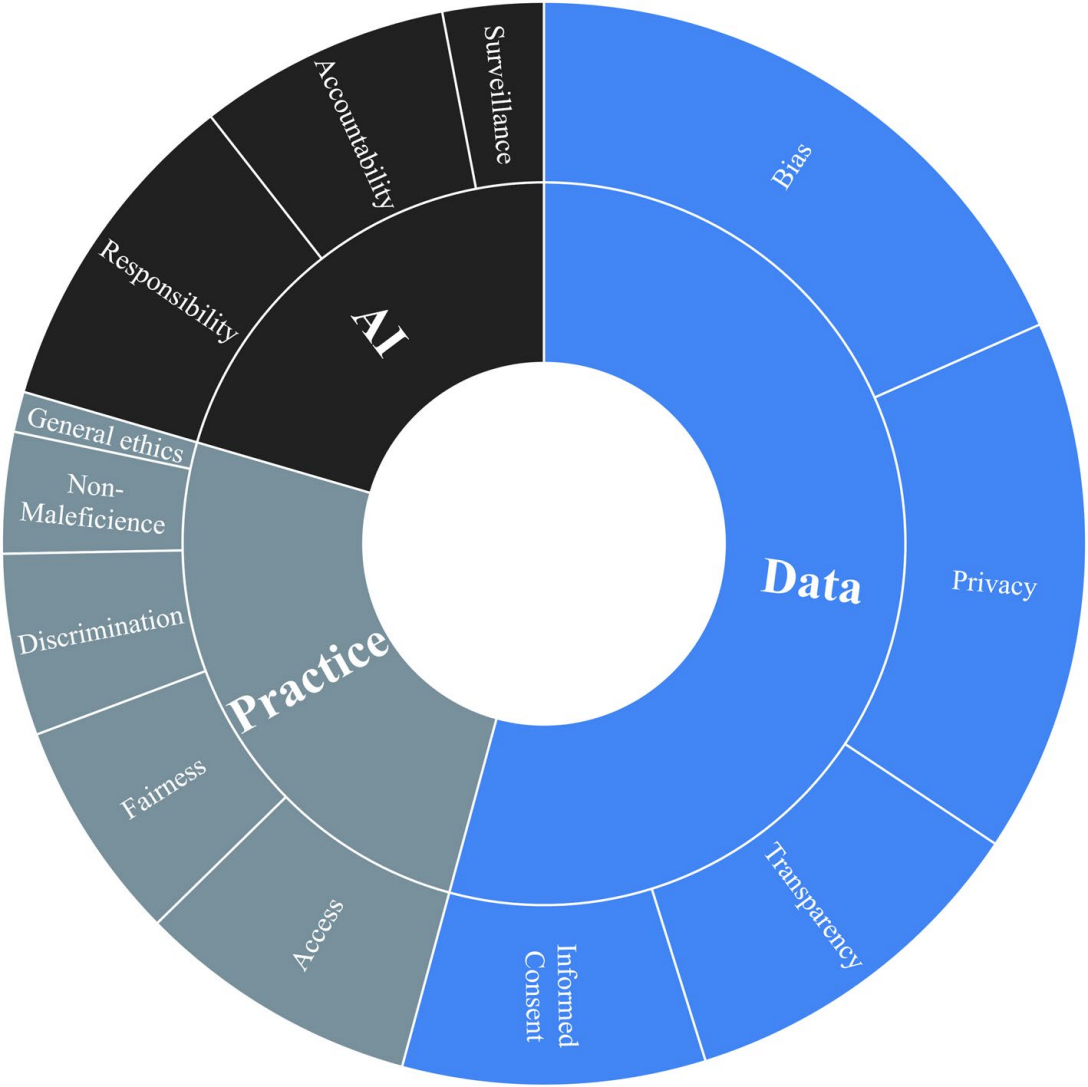
Ethical Considerations from the Literature. Data collected by A. Itani, S. Gronseth, S. Musaad, T. Nguyen, and Y. Mirabile in 2023 from PubMed, Embase, and Web of Science

Ethical considerations discussed in the articles span data use, algorithm applications, and how algorithms are applied to practice (see Fig. 4). Ethical issues relating to data use were most frequently mentioned, with 74% of the articles discussing bias, 65% privacy, 44% transparency, and 37% informed consent. Ethical considerations relating to algorithm applications included responsibility (40%), accountability (30%), and surveillance (12%). For how algorithms are applied to practice, articles mentioned access (34%), fairness (27%), discrimination (22%), non-maleficence (15%), and general ethics (5%) concerns. A chi-square test of independence was conducted to examine the relationship between ethical considerations and article focus areas. The test revealed no statistically significant association between the ethical considerations and the primary focus of the articles, χ^2^(16) = 14.37, *p* = 0.98.

Furthermore, articles discussing privacy concerns (65%) emphasized the need for robust data protection measures, particularly in AI tools used for diagnostic simulations. Several studies (e.g., Busch et al., 2023; Sqalli et al., 2023) noted that students must be trained in navigating these issues to build trust in AI systems and prevent the misuse of patient data. Algorithmic bias was frequently linked to disparities in AI-generated diagnoses for underrepresented populations. Articles by Ejaz et al. (2022) and Paranjape et al. (2019) proposed incorporating bias detection and mitigation strategies into medical education curricula to address these concerns.

## Discussion

### Key findings from the reviewed articles

This review explored publication patterns of literature on AI ethical considerations in medical education settings. In this section, we will highlight key findings from the corpus of articles included in this scoping review and offer a synthesis of the observed primary themes. There has been a noticeable increase in publications on this topic since 2022 by authors from around the globe. This rise in recent years signals increasing awareness of AI’s ethical implications in medical education, driven by its rapid integration into training. Designing AI systems in medical education necessitates transparency, fairness and justice, safety and well-being, accountability, and collaboration (Köbis & Mehner, 2021; Nguyen et al., 2023; Sqalli et al., 2023). The pressing need to develop ethical frameworks and guidelines for the responsible integration of AI in education is evident. Further, the broad distribution of articles across numerous journals indicates that scholarly discourse on this subject is taking place in many professional spaces.

The literature is interdisciplinary, encompassing diverse fields of academic medicine, education, artificial intelligence, and ethics. The prevalence of review articles suggests the field remains largely exploratory, focusing more on synthesizing existing knowledge and less on conducting empirical studies. This indicates a gap in direct research on AI’s impact, emphasizing the need for future studies to assess instructional methods, student experiences, and ethical challenges in practice. While literature reviews were the most frequent article type, empirical research on AI ethics in medical education seems to be expanding. There are indications of the embedding of the perspectives and knowledge of medical professionals/academicians, computer and data science professionals/academics, law and ethics professionals/academics, and medical students into the development of curricula on the ethical use of AI in medicine (Çalışkan et al., 2022; Wood et al., 2021). Interdisciplinary research on the instructional design and evaluation of ethically grounded, AI-integrated instruction in medical education settings is needed (Weidener & Fischer, 2023). Next, we will discuss the three primary focus areas observed in the articles.

*Incorporating AI in Medical Education*. Lee et al. (2021) mention that despite growing interest in emerging technologies, medical education has struggled to keep up with the rapid advancements in AI. There have been numerous calls for action, yet the integration of AI into undergraduate medical education has been thus far minimal, likely due to the absence of comprehensive evidence. One way to ensure beneficence is to offer structured training on AI tools before their integration into medical education (Grunhut et al., 2022; Paranjape et al., 2019). However, many educators lack the technical expertise to effectively teach AI-related content, emphasizing the need for professional development programs tailored to building AI literacy (Çalışkan et al., 2022). Institutional resistance to curriculum changes and insufficient policy support for ethical AI training further complicate efforts to embed these concepts meaningfully into existing educational frameworks (Wood et al., 2021).

To address these challenges, medical school curricula should be updated to prioritize AI proficiency, knowledge management, and the development of communication skills and empathy (Wartman & Combs, 2019). Several authors emphasize the importance of incorporating AI ethical principles into medical education while advocating for innovative teaching methods and ongoing evaluation to ensure effective integration (e.g., Chan & Zary, 2019). The goal is to train the next generation of physicians to collaborate with AI while upholding core values in patient-centered care.

The literature highlights the importance of transparency in AI limitations and biases to preserve user autonomy and mitigate potential harm (Cobianchi et al., 2022). Some recommendations include involving stakeholders in decision-making, ensuring equitable access to AI tools, and regularly auditing AI applications for biases (Sqalli et al., 2023). Additionally, the absence of standardized ethical frameworks for AI in medical education poses a significant challenge to responsible implementation. Holmes et al. (2021) stress that ethical intentions alone are insufficient, and structured guidelines are necessary to address fairness, accountability, transparency, and bias in AI-powered educational tools. Without proper training, educators may inadvertently rely on AI systems that reinforce biases, compromise data privacy, or create inequitable learning experiences.

*AI Ethical Principles in Medical Education*. Topics of bias, privacy, and transparency were frequently explored by authors, highlighting the need for rigorous safeguards and policies to ensure fairness in AI-driven educational tools. Designing a curriculum to address AI-related challenges requires a strong emphasis on ethical AI literacy and critical evaluation of these technologies (Alam et al., 2023). Providing hands-on experience with AI-powered instructional tools, such as virtual patient simulations and automated grading systems, allows learners to assess how AI functions and identify potential biases (Abd-alrazaq et al., 2023). Also, collaboration between medical educators, AI developers, and ethicists can be helpful in developing guidelines for the responsible use of AI in education that fosters transparency and accountability (Busch et al., 2023). Embedding explainability features in AI tools and ensuring diverse datasets for AI-generated content can enhance fairness and reliability.

Additionally, to address issues of algorithmic bias and the broader implications of AI-driven healthcare disparities, dedicated modules on AI ethics that incorporate case studies illustrating real-world scenarios of bias in medical AI applications are recommended (Nguyen et al., 2023). Interactive workshops could explore how biased datasets in diagnostic tools may disproportionately affect minority populations, prompting discussions on mitigating such risks through algorithm design and oversight (Sallam, 2023). Small group discussions and role-playing activities can help students critically analyze ethical dilemmas related to AI transparency and accountability (Sqalli et al., 2023).

*ChatGPT in Healthcare Education*. AI tools like ChatGPT are fueling the rethinking of assessment strategies and teaching methods as they provide expanded options in content representation and knowledge building (Abd-alrazaq et al., 2023; Rahimzadeh et al., 2023). Such resulting changes in curriculum design, assessment strategies, and teaching methods can lead to educators adapting their teaching approaches to incorporate instructional technology and ensure that students develop necessary digital literacy skills (Lee, 2023; Schukow et al., 2024). In the articles reviewed, application examples included using ChatGPT as a point-of-need resource for medical students, generating self-check quizzes, and incorporating it as part of interactive simulations; these uses enriched learning experiences and bridged the gap between theory and practice (e.g., Eysenbach, 2023).

ChatGPT can significantly expedite literature reviews, enhance writing efficiency, aid in generating computer codes, and streamline workflows (Dave et al., 2023; Wang et al., 2023). Additionally, it has promising implications for improving diagnostics, advancing personalized medicine, providing personalized learning experiences, generating efficient educational content, and offering opportunities for interactive learning (Kung et al., 2023). However, the authors highlighted limitations and ethical considerations regarding accuracy, bias, transparency, and plagiarism (Dossantos et al., 2023; Sallam, 2023). Moving forward, responsible and ethical applications of ChatGPT and other generative AI tools are likely to be supported through the development of guidelines and ongoing evaluation to maximize benefits while mitigating risks (Dave et al., 2023; Wang et al., 2023).

### Strategies for addressing ethical considerations

The integration of AI into medical education has significant challenges related to data privacy, transparency, and algorithmic bias (Grunhut et al., 2022; Paranjape et al., 2019). AI-powered learning tools collect vast amounts of user data, raising concerns about data protection and ethical use (Hanna et al., 2024; Sheffield et al., 2024). To address these issues, institutions are recommended to implement strong governance policies, including data anonymization, clear consent protocols, and transparency in AI decision-making (De et al., 2023). Compliance with regulations such as the General Data Protection Regulation (GDPR) and the Health Insurance Portability and Accountability Act (HIPAA) is essential to ensure data security and ethical management (Grunhut et al., 2022). Additionally, updating ethics approval processes and guidelines is necessary to address AI’s impact on data privacy and ethics in education, aligning institutional policies with programmatic goals and ethical standards (Nguyen et al., 2023; Satapathy et al., 2023).

Transparency in how AI is being used in medical education presents significant challenges. AI-powered tools such as grading systems and diagnostic models often operate using opaque algorithms, making it difficult for students and educators to understand how decisions are made (Ramnani, 2024). This lack of transparency calls into question accountability and trust, particularly when AI-generated recommendations influence educational assessments and learning outcomes. To enhance transparency, faculty training programs could help educators interpret AI-generated outputs, while student workshops could foster AI literacy and critical evaluation skills (Flores-Vivar & García-Peñalvo, 2023; Memarian & Doleck, 2023). These strategies could help users understand the limitations and ethical implications of AI-driven recommendations.

The most frequently mentioned ethical consideration, bias, is at the forefront of ethical AI discussions and guidelines from organizations like the AMA (2019). These guidelines emphasize that bias reduction is critical in integrating AI tools in medical education, as biased algorithms can reinforce disparities in clinical training and decision-making (Hanna et al., 2024). Bias in AI-generated educational content and assessments may disproportionately affect certain student groups, reinforcing existing disparities (Busch et al., 2023). AI-powered grading and diagnostic tools may contribute to inequitable outcomes if training datasets are unrepresentative (De et al., 2023). Regular algorithmic audits are necessary to detect and correct biases in AI systems (Flores-Vivar & García-Peñalvo, 2023), while diverse and representative datasets can help support more equitable AI outcomes (Ramnani, 2024). Educators can mitigate these risks by selecting AI-powered tools with built-in explainability features that clarify how the AI-generated recommendations are made (Sqalli et al., 2023). Additionally, integrating fairness literacy into medical curricula can equip students with the skills to identify and mitigate bias through real-world case studies (Memarian & Doleck, 2023).

By comprehensively addressing privacy, transparency, bias, and ethical education, medical education institutions worldwide can take proactive steps to infuse AI ethically into their medical training experiences. Different approaches have been adopted, ranging from macro-ethics, such as cyberspace regulation, to micro-ethics, such as teamwork and professional conduct (Memarian & Doleck, 2023). For example, AI literacy programs in Hong Kong aim to equip students with ethical awareness and critical thinking skills (Memarian & Doleck, 2023). Addressing these challenges requires embedding AI ethics courses within medical education to prepare students for ethical dilemmas in AI-driven healthcare (Sheffield et al., 2024), promoting interdisciplinary collaboration between medical and AI professionals to ensure responsible AI development (Flores-Vivar & García-Peñalvo, 2023), and encouraging faculty and students to actively engage in discussions on AI ethics, fostering a culture of ethical awareness and critical evaluation (Ramnani, 2024).

### Limitations

While we strived to conduct this scoping review with the utmost precision, the review is limited to articles indexed in three databases and published in English. Future reviews could build upon this work to surface insights from articles published in other languages. This limitation may skew ethical perspectives, potentially excluding culturally diverse viewpoints and region-specific challenges. The qualitative interpretations of the ethical issues addressed in the articles are subjective and open to other possible interpretations. Further, AI technologies and AI-related publications about ethical considerations in medical education are accelerating; thus, the findings from this review will likely continue to grow and expand in the coming years. We acknowledge that these limitations may have impacted the results by potentially narrowing the scope of included perspectives and emerging insights, which could influence the generalizability and relevance of the findings over time.

## Conclusions and implications for future educational research

The increasing integration of AI in medical education requires efforts to ensure that AI systems align with ethical principles and promote equitable outcomes (Sqalli et al., 2023). There is an expansion of scholarly information on using AI in medical education settings, and educators are grappling with how to effectively integrate these AI tools into their curricula and instructional practices (Celik et al., 2022). Review, editorial, and empirical articles largely comprise the discourse, particularly on the themes of incorporating AI in medical education and its challenges. Theoretical articles are starting to emerge, with opportunities for theoretical perspectives on related conceptual frameworks. Future research is needed to develop comprehensive theories that underpin AI’s effective and ethical integration in medical education.

There are also needs for more empirical research on student perceptions and ongoing dialogues with diverse stakeholders on adapting to evolving AI technologies while accounting for the ethical implications of such rapid changes. If medical institutions leverage open-source AI education platforms, free or low-cost AI-driven tutoring, simulation, and assessment tools could be provided to promote equitable access to AI-enhanced learning tools. Additionally, partnerships with AI developers could ensure that students in resource-limited settings can access the same advanced AI-based learning experiences as students in well-funded institutions (Ejaz et al., 2022). Future research should include longitudinal studies that assess how AI ethics training shapes students' ability to critically evaluate and responsibly use AI-driven educational tools in medical learning. These studies could track how well students retain AI ethics knowledge, apply fairness and transparency principles in AI-assisted instruction, and develop trust in AI-generated assessments and learning materials.

The development and refinement of regulatory and ethical frameworks for responsible AI use are essential to mitigating unintended consequences of its utilization (Saheb et al., 2021). The ethical implications of how algorithms are applied, such as issues of responsibility, accountability, and fairness, highlight the importance of developing AI systems that are not only practical but also ethically sound and equitable (Olorunsogo et al., 2024). Addressing these considerations can foster the responsible integration of AI with the support of student and faculty trust in using AI systems. Recommended ways to teach these topics are through interactive seminars and small group discussions using real-life examples (Weidener & Fischer, 2023). Further work on consensus-building toward standardized definitions and measures for ethics in AIEd will aid in promoting responsible and ethical uses of AI technologies as part of instructional practices.

Many educators are concerned about confidentiality, informed consent, and data security (Hanna et al., 2024). This scoping review offers a foundation for future studies that could expand these lines of inquiry. Ethical frameworks emphasize the need for strong data governance, anonymization measures, and restricted access to prevent unauthorized use of patient information in AI-driven training tools and simulations (Nasir et al., 2024). To further advance the field, collaboration among educators, ethicists, and AI experts is crucial in developing a shared educational approach that ensures AI technologies are leveraged ethically and equitably. Interdisciplinary collaborations could pave the way for innovations in teaching with AI ethically, such as AI-supported case-based learning and simulation-based training. Opportunities abound in developing standardized metrics for assessing ethical practices in AI-integrated education, ensuring that guidelines are actionable and widely applicable across diverse medical education contexts.

## Data Availability

The datasets used and/or analyzed during the current study (the bibliography of included studies) are available from the corresponding author upon request. Appendix A summarizes all the included articles in the study, listing authors, titles, publication years, journals, countries, research focus, ethical considerations, and relevant themes.

## Appendix A: Complete list of included articles

**Table:**

#	Authors	Title	Type^1^	Year	Journal	Country	Focus	Ethical considerations	Theme
1	Abd-alrazaq et al	Large language models in medical education: Opportunities, challenges, and future directions	Ed	2023	*JMIR Medical Education*	Qatar	Data lifecycle and ethics in surgical research	Bias, fairness, privacy, informed consent, responsibility, access	AI Ethical Principles in Medical Education
2	Abdellatif et al	Teaching, learning and assessing anatomy with artificial intelligence: The road to a better future	R	2022	*International Journal of Environmental Research and Public Health*	Oman	Ethical and educational implications of AI in surgery	Access	Challenges (and Opportunities) in AI Medical Education
3	Abdullah et al	Ethics of artificial intelligence in medicine and ophthalmology	R	2021	*Asia–Pacific Journal of Ophthalmology*	United States	AI and ethics in medical education: current and future issues	Privacy, informed consent, transparency, accountability, responsibility, non-maleficence	AI Ethical Principles in Medical Education
4	Albahri et al	A systematic review of trustworthy and explainable artificial intelligence in healthcare: Assessment of quality, bias risk, and data fusion	R	2023	*Information Fusion*	Iraq	Trustworthiness and explainability of AI in healthcare	Bias, discrimination, fairness, privacy, informed consent, transparency, responsibility, access	Incorporating AI in Medical Education
5	Alkhulaifat et al	Implications of pediatric artificial intelligence challenges for artificial intelligence education and curriculum development	R	2023	*Journal of the American College of Radiology*	United States	AI in medical education: challenges, benefits, and future trends	Bias, privacy, informed consent, responsibility	AI Competency
6	Alqahtani et al	The emergent role of artificial intelligence, natural learning processing, and large language models in higher education and research	R	2023	*Research in Social and Administrative Pharmacy*	Saudi Arabia	ChatGPT’s applications and limitations in ophthalmology	Bias, privacy, transparency	Evaluation of Medical AI Readiness
7	Arora & Arora	Generative adversarial networks and synthetic patient data: Current challenges and future perspectives	Ed	2022	*Future Healthcare Journal*	United Kingdom	Evaluation of AI readiness scales for medical students	Bias, privacy	Incorporating AI in Medical Education
8	Aulenkamp et al	Overview of digital health teaching courses in medical education in Germany in 2020	Em	2021	*GMS Journal for Medical Education*	Germany	Medical students' perspectives on AI's role in medicine	Access	AI Competency
9	Barreiro-Ares et al	Impact of the rise of artificial intelligence in radiology: What do students think?	Em	2023	*International Journal of Environmental Research and Public Health*	Spain	AI’s impact on medical education reform and curriculum changes	Privacy	AI Case-Based Learning
10	Bilgic et al	Exploring the roles of artificial intelligence in surgical education: A scoping review	R	2021	*The American Journal of Surgery (AJS)*	Canada	Enhancing healthcare with AI and addressing ethical challenges	General ethics	Incorporating AI in Medical Education
11	Blease et al	Machine learning in clinical psychology and psychotherapy education: A mixed methods pilot survey of postgraduate students at a Swiss university	Em	2021	*Frontiers in Public Health*	United States	Overview of AI’s role and effectiveness in healthcare education	Bias, privacy	AI Ethical Principles in Medical Education
12	Briganti & Le Moine	Artificial Intelligence in Medicine: Today and Tomorrow	R	2020	*Frontiers in Medicine*	Belgium	Benefits, opportunities, and risks of AI in clinical practice	Bias, privacy	Incorporating AI in medical education
13	Busch et al	Biomedical ethical aspects towards the implementation of artificial intelligence in medical education	Ed	2023	*Medical Science Educator*	Germany	ChatGPT’s use in diabetes education: benefits and limitations	Bias, discrimination, fairness, privacy, informed consent, transparency, accountability, non-maleficence	AI Ethical Principles in Medical Education
14	Çalışkan et al	Artificial intelligence in medical education curriculum: An e-Delphi study for competencies	Em	2022	*PLoS ONE*	Turkey	AI in medical education: current challenges and future outlook	Responsibility	Evaluation of Medical AI Readiness
15	Chamunyonga et al	The Impact of Artificial Intelligence and Machine Learning in Radiation Therapy: Considerations for Future Curriculum Enhancement	R	2020	*Journal of Medical Imaging and Radiation Sciences*	Australia	AI and ML in radiation therapy and educational integration	Bias, data privacy, informed consent, accountability	Incorporating AI in Medical Education
16	Chan & Zary	Applications and challenges of implementing artificial intelligence in medical education: Integrative review	R	2019	*JMIR Medical Education*	Singapore	Ethical issues of AI in health from a global perspective	Privacy, access	Incorporating AI in Medical Education
17	Charow et al	Artificial Intelligence Education Programs for Health Care Professionals: Scoping Review	R	2021	*JMIR Medical Education*	Canada	AI’s impact on academic authorship and ethics	Bias, privacy, transparency, responsibility, access	Evaluation of Medical AI Readiness
18	Chaudhry & Kazim	Artificial intelligence in education (AIEd): A high-level academic and industry note 2021	R	2021	*AI and Ethics*	United Kingdom	AI's impact on curriculum and medical education	Bias, discrimination, fairness, privacy, informed consent, transparency, responsibility	AI Ethical Principles in Medical Education
19	Civaner et al	Artificial intelligence in medical education: A cross-sectional needs assessment	Em	2022	*BMC Medical Education*	Turkey	Integration of AI in medical education: benefits and barriers	Privacy, informed consent	AI Competency
20	Cobianchi et al	Artificial intelligence and surgery: Ethical dilemmas and open issues	Em	2022	*Journal of the American College of Surgeons*	United States	Impact of AI on medical curriculum development	Bias, privacy, informed consent, transparency, accountability, responsibility, discrimination, fairness	Incorporating AI in Medical Education
21	Combs & Combs	Emerging roles of virtual patients in the age of AI	R	2019	*AMA Journal of Ethics*	United States	Benefits and challenges of AI in medical education	Bias, fairness, responsibility	AI Ethical Principles in Medical Education
22	Dave et al	ChatGPT in medicine: an overview of its applications, advantages, limitations, future prospects, and ethical considerations	R	2023	*Frontiers in Artificial Intelligence*	Ukraine	The future of AI in medical education and research	Bias, discrimination, transparency, responsibility, accountability	Incorporating AI in Medical Education
23	Dossantos et al	Eyes on AI: ChatGPT's transformative potential impact on ophthalmology	Ed	2023	*Cureus*	United States	ChatGPT’s potential as an AI tool in medical education	Bias, privacy, transparency	ChatGPT in Healthcare Education
24	Eckhoff et al	SAGES consensus recommendations on surgical video data use, structure, and exploration (for research in artificial intelligence, clinical quality improvement, and surgical education)	Em	2023	*Surgical Endoscopy*	United States	Exploring ChatGPT’s applications and effectiveness in medicine	Bias, fairness privacy, informed consent, responsibility	Incorporating AI in Medical Education
25	Ejaz et al	Artificial intelligence and medical education: A global mixed-methods study of medical students' perspectives	Em	2022	*Digital Health*	United Kingdom	AI in education: practical applications and limitations	Bias, discrimination, access	AI Training in Medical Education
26	Ellaway and Tolsgaard	Artificial scholarship: LLMs in health professions education research	Ed	2023	*Advances in Health Sciences Education*	Canada	ChatGPT’s potential and limitations in medical education	Bias, accountability	AI Ethical Principles in Medical Education
27	Eysenbach	The role of ChatGPT, generative language models, and artificial intelligence in medical education: A conversation with ChatGPT and a call for papers	Ed	2023	*JMIR Medical Education*	Canada	Current issues in AI and medical education	Bias, privacy, transparency	ChatGPT in Healthcare Education
28	Gray et al	Artificial intelligence education for the health workforce: Expert survey of approaches and needs	Em	2022	*JMIR Medical Education*	Australia	Experts' views on AI curriculum gaps in healthcare	Bias, access	Incorporating AI in Medical Education
29	Grunhut et al	Needs, challenges, and applications of artificial intelligence in medical education curriculum	Ed	2022	*JMIR Medical Education*	United States	Challenges of AI in healthcare and educational contexts	Transparency, accountability, access	Incorporating AI in Medical Education
30	Holmes et al	Ethics of AI in education: Towards a community-wide framework	Em	2021	*International Journal of Artificial Intelligence in Education*	United Kingdom	Challenges and prospects of AI in health education	Bias, fairness, privacy, informed consent, transparency, responsibility, access, surveillance, non-maleficence	Incorporating AI in Medical Education
31	Huisman et al	An international survey on AI in radiology in 1041 radiologists and radiology residents part 2: Expectations, hurdles to implementation, and education	Em	2021	*European Radiology*	Netherlands	Ethical issues with AI in education	Bias, privacy	Evaluation of Medical AI Readiness
32	James et al	Machine learning: The next paradigm shift in medical education	Ed	2021	*Academic Medicine*	United States	AI’s role in modernizing medical education and training	Bias, accountability, responsibility	AI Ethical Principles in Medical Education
33	Karabacak et al	The advent of generative language models in medical education	Ed	2023	*JMIR Medical Education*	United States	Evaluating AI’s role in surgical education and training	Bias, discrimination, privacy, transparency, fairness. Responsibility, access, non-maleficence	Challenges (and Opportunities) in AI Medical Education
34	Karaca et al	Medical artificial intelligence readiness scale for medical students (MAIRS-MS)—development, validity and reliability study	Em	2021	*BMC Medical Education*	Turkey	Key ethical issues in AI for healthcare education	General ethics	AI Ethical Principles in Medical Education
35	Katznelson & Gerke	The need for health AI ethics in medical school education	Ed	2021	*Advances in Health Sciences Education*	Canada	Overview of AI education programs: content, delivery, outcomes	Bias, privacy, informed consent, transparency, responsibility, access, non-maleficence	Incorporating AI in Medical Education
36	Köbis & Mehner	Ethical questions raised by AI-supported mentoring in higher education	R	2021	*Frontiers in Artificial Intelligence*	Germany	Digital competencies in medical curricula and their importance	Bias, discrimination, fairness, privacy, informed consent, transparency, accountability, surveillance, non-maleficence	AI Ethical Principles in Medical Education
37	Kolachalama & Garg	Machine learning and medical education	Ed	2018	*npj Digital Medicine*	United States	AI’s potential in medical training and practical applications	Bias	Incorporating AI in Medical Education
38	Kolachalama	Machine learning and pre-medical education	Ed	2022	*Artificial Intelligence in Medicine*	United States	AI’s role in improving medical education and practice	Privacy	AI Training in Medical Education
39	Kung et al	Performance of ChatGPT on USMLE: Potential for AI-assisted medical education using large language models	Em	2023	*PLoS Digital Health*	United States	AI’s impact on medical student training and curriculum	Bias, privacy, informed consent	ChatGPT in Healthcare Education
40	Lazarus et al	Artificial intelligence and clinical anatomical education: Promises and perils	R	2022	*Anatomical Sciences Education*	Australia	Tensions in integrating AI into anatomy education	Bias, discrimination, privacy, informed consent, transparency, surveillance	AI Ethical Principles in Medical Education
41	Lee	The rise of ChatGPT: Exploring its potential in medical education	Ed	2023	*Anatomical Sciences Education*	Republic of Korea	Traditional ethics education vs. AI competencies in training	Bias	Incorporating AI in Medical Education
42	Lee et al	Artificial intelligence in undergraduate medical education: A scoping review	R	2021	*Academic Medicine*	Canada	Roles, theories, and strategies for AI in education	Bias, privacy, informed consent, transparency, responsibility	AI Ethical Principles in Medical Education
43	Malerbi et al	Digital education for the deployment of artificial intelligence in health care	Ed	2023	*Journal of Medical Internet Research*	Brazil	Gaps in AI training in undergraduate medical education	Bias, fairness, privacy, informed consent	Incorporating AI in Medical Education
44	Masters	Artificial intelligence in medical education	R	2019	*Medical Teacher*	Oman	Challenges in AI training for radiology and proposed solutions	Responsibility	Incorporating AI in Medical Education
45	Masters	Ethical use of artificial intelligence in health professions education: AMEE Guide No.158	R	2023	*Medical Teacher*	Oman	Defining AI competencies for healthcare professionals	Bias, discrimination, fairness, privacy, informed consent, transparency, responsibility, access, surveillance, non-maleficence	AI Ethical Principles in Medical Education
46	Masters	The global use of artificial intelligence in the undergraduate medical curriculum: A systematic review	R	2023	*Cureus*	Oman	Differences in AI models for pediatric versus adult practice	Bias, fairness, privacy, informed consent, transparency, accountability, responsibility, surveillance, access, non-maleficence	Challenges (and Opportunities) in AI Medical Education
47	McLennan et al	German medical students´ views regarding artificial intelligence in medicine: A cross-sectional survey	Em	2022	*PLoS Digital Health*	Germany	Ethical challenges of integrating AI into medical education	Bias, discrimination, privacy, transparency, accountability, responsibility	Evaluation of Medical AI Readiness
48	Mohammed et al	Psychometric properties and assessment of knowledge, attitude, and practice towards ChatGPT in pharmacy practice and education: A study protocol	Em	2023	*Journal of Racial and Ethnic Health Disparities*	Nigeria	Need for AI education reform to meet healthcare demands	Bias, privacy, access	ChatGPT in Healthcare Education
49	Morrow et al	Artificial intelligence technologies and compassion in healthcare: A systematic scoping review	R	2023	*Frontiers in Psychology*	Switzerland	Exploring AI’s role in medical education and student engagement	Bias	Evaluation of Medical AI Readiness
50	Murphy et al	Artificial intelligence for good health: A scoping review of the ethics literature	R	2021	*BMC Medical Ethics*	Canada	Exploring ethical and practical issues in AI-based education	Bias, discrimination, fairness, privacy, informed consent, transparency, accountability, responsibility, surveillance, access	Challenges (and Opportunities) in AI Medical Education
51	Nagy et al	Why machine learning should be taught in medical schools	Ed	2022	*Medical Science Educator*	United States	Impact of AI on medical education and professional training	Bias, responsibility, transparency	Incorporating AI in Medical Education
52	Nguyen et al	Ethical principles for artificial intelligence in education	T	2023	*Education and Information Technologies*	Finland	Ethical questions in AI-supported mentoring environments	Bias, discrimination, fairness, privacy, informed consent, transparency, accountability, responsibility, surveillance, access, non-maleficence	AI Ethical Principles in Medical Education
53	Ossa et al	A smarter perspective: Learning with and from AI-cases	T	2022	*Artificial Intelligence in Medicine*	Switzerland	Trustworthiness and ethics of AI in medical practice	Bias, informed consent, accountability	Incorporating AI in Medical Education
54	Paranjape et al	Introducing artificial intelligence training in medical education	Ed	2019	*JMIR Medical Education*	Netherlands	AI techniques for teaching anatomy and lessons learned	Bias, privacy, informed consent, transparency access	Incorporating AI in Medical Education
55	Perchik et al	Artificial intelligence literacy: Developing a multi-institutional infrastructure for AI education	Em	2022	*Academic Radiology*	United States	Ethical implications of AI use in medical education	Privacy, transparency	Incorporating AI in Medical Education
56	Quinn et al	Trust and medical AI: The challenges we face and the expertise needed to overcome them	Ed	2021	*Journal of the American Medical Informatics Association*	United States	Evaluating the effectiveness of AI in healthcare education	Privacy, informed consent, transparency, responsibility, non-maleficence	Challenges (and Opportunities) in AI Medical Education
57	Rahimzadeh et al	Ethics education for healthcare professionals in the era of ChatGPT and other large language models: Do we still need it?	T	2023	*The American Journal of Bioethics*	United States	AI's impact on ethical standards in medical education	Bias	ChatGPT in Healthcare Education
58	Rezazadeh et al	Psychometric evaluation of Persian version of medical artificial intelligence readiness scale for medical students	Em	2023	*BMC Medical Education*	Iran	AI in medical education: current state and future directions	General ethics	Evaluation of Medical AI Readiness
59	Roy et al	Analyzing the evolution of medical ethics education: A bibliometric analysis of the top 100 cited articles	R	2023	*Cureus*	United Kingdom	AI’s role in enhancing medical education and practice	General ethics	AI Ethical Principles in Medical Education
60	Russell et al	Competencies for the use of artificial intelligence-based tools by health care professionals	Em	2022	*Academic Medicine*	United States	Ethical implications of AI in medical practice and education	Responsibility, fairness, bias	AI Ethical Principles in Medical Education
61	Sallam	ChatGPT utility in healthcare education, research, and practice: Systematic review on the promising perspectives and valid concerns	R	2023	*Healthcare*	Jordan	AI’s impact on education: benefits, challenges, and ethics	Bias, discrimination, fairness, privacy, transparency, responsibility, access	ChatGPT in Healthcare Education
62	Santana et al	Combining ChatGPT and machine learning: A viable alternative for discussion in oral medicine	Ed	2023	*Oral Diseases*	Brazil	Importance of teaching digital health for AI benefits	Bias	Incorporating AI in Medical Education
63	Sapci & Sapci	Artificial intelligence education and tools for medical and health informatics students: Systematic review	R	2020	*JMIR Medical Education*	United States	Ethical challenges of integrating AI into medical curricula	Bias, surveillance	Incorporating AI in Medical Education
64	Satapathy et al	Artificial intelligence in surgical education and training: Opportunities, challenges, and ethical considerations—correspondence	Ed	2023	*International Journal of Surgery*	India	Evaluating AI readiness in medical students	Bias, discrimination, fairness, privacy, informed consent, transparency, responsibility	Challenges (and Opportunities) in AI Medical Education
65	Schiff	Education for AI, not AI for education: The role of education and ethics in national AI policy strategies	Em	2021	*International Journal of Artificial Intelligence in Education*	United States	Exploring ChatGPT’s potential in medical diagnostics	Bias, fairness, privacy, informed consent, transparency, responsibility, access, non-maleficence	Incorporating AI in Medical Education
66	Schukow et al	Application of ChatGPT in routine diagnostic pathology: Promises, pitfalls, and potential future directions	R	2024^1^	*Advances In Anatomic Pathology*	United States	Evaluating global consensus on AI in healthcare education	Bias, privacy, transparency, accountability	Incorporating AI in Medical Education
67	Senbekov et al	The recent progress and applications of digital technologies in healthcare: A review	R	2020	*International Journal of Telemedicine and Applications*	Kazakhstan	AI’s influence on medical education and practice	Bias, discrimination, privacy, responsibility, access	Incorporating AI in Medical Education
68	Sharma et al	A critical review of ChatGPT as a potential substitute for diabetes educators	R	2023	*Cureus*	India	Opportunities and challenges of AI in surgery education	Bias, discrimination, privacy, informed consent	ChatGPT in Healthcare Education
69	Sqalli et al	Humanizing AI in medical training: Ethical framework for responsible design	T	2023	*Frontiers in Artificial Intelligence*	Qatar	ChatGPT’s potential in diagnostic pathology and future use	Bias, fairness, privacy, transparency, responsibility, accountability	ChatGPT in Healthcare Education
70	Sun et al	Artificial intelligence for healthcare and medical education: A systematic review	R	2023	*American Journal of Translational Research*	China	ChatGPT’s effectiveness and challenges in medical settings	Privacy, informed consent	Incorporating AI in Medical Education
71	Tadavarthi et al	Overview of noninterpretive artificial intelligence models for safety, quality, workflow, and education applications in radiology practice	R	2022	*Radiology: Artificial Intelligence*	United Kingdom	Addressing ethical concerns in AI-based medical education	Surveillance	AI Student Knowledge Perspectives
72	Tejani et al	Artificial intelligence and radiology education	Ed	2022	*Radiology: Artificial Intelligence*	United States	AI’s role in shaping future medical education practices	Bias, access, privacy	Incorporating AI in Medical Education
73	Thoma et al	From utopia through dystopia: Charting a course for learning analytics in competency-based medical education	T	2021	*Academic Medicine*	United States	Analysis of AI's integration into medical education	Bias, privacy, informed consent, transparency, access	AI Ethical Principles in Medical Education
74	Tulgar et al	Anesthesiologists' perspective on the use of artificial intelligence in ultrasound-guided regional anaesthesia in terms of medical ethics and medical education: A survey study	Em	2023	*The Eurasian Journal of Medicine*	Turkey	Ethical implications of AI in medical education	Privacy, informed consent	AI Ethical Principles in Medical Education
75	van de Venter et al	Artificial intelligence education for radiographers, an evaluation of a UK postgraduate educational intervention using participatory action research: A pilot study	Em	2023	*Insights into Imaging*	United Kingdom	AI’s influence on medical education and ethical considerations	Bias, privacy, transparency, responsibility	ChatGPT in Healthcare Education
76	Waller et al	Applications and challenges of artificial intelligence in diagnostic and interventional radiology	R	2022	*Polish Journal of Radiology*	United States	Exploring ethical frameworks for AI in medical education	Transparency, accountability, responsibility	Incorporating AI in Medical Education
77	Wang et al	Performance and exploration of ChatGPT in medical examination, records and education in Chinese: Pave the way for medical AI	Em	2023	*International Journal of Medical Informatics*	China	Global consensus on ethical AI in education	Transparency, accountability	Evaluation of Medical AI Readiness
78	Wartman & Combs	Medical education must move from the information age to the age of artificial intelligence	Ed	2018	*Academic Medicine*	United States	The future of AI in health education and research	Accountability, surveillance	Incorporating AI in Medical Education
79	Wartman & Combs	Reimagining medical education in the age of AI	Ed	2019	*AMA Journal of Ethics*	United States	Ethical considerations of AI applications in medicine	Bias, responsibility	Incorporating AI in Medical Education
80	Weidener & Fischer	Artificial intelligence teaching as part of medical education: Qualitative analysis of expert interviews	Em	2023	*JMIR Medical Education*	Austria	Understanding AI’s role and curriculum in medical education	Bias, discrimination, privacy, informed consent, transparency, surveillance	Incorporating AI in Medical Education
81	Wood et al	Are we ready to integrate artificial intelligence literacy into medical school curriculum: Students and faculty survey	Em	2021	*Journal of Medical Education and Curricular Development*	United States	AI’s potential in radiology education and practice	General ethics	AI Student Knowledge Perspectives
82	Zhong & Fischer	Commentary: The desire of medical students to integrate artificial intelligence into medical education: An opinion article	Ed	2023	*Frontiers in Digital Health*	Singapore	Evaluating AI literacy course effectiveness in radiology	Bias	Incorporating AI in Medical Education

^a^This article was available as an advanced Epub on July 27, 2023.

## Abbreviations

AI: Artificial intelligence
PRISMA-ScR: Preferred Reporting Items for Systematic reviews and Meta-Analyses extension for Scoping Reviews
